# Enlarging the clinical spectrum of SAVI syndrome

**DOI:** 10.1186/1546-0096-13-S1-P70

**Published:** 2015-09-28

**Authors:** R Caorsi, G Rice, S Volpi, F Cardinale, A Buoncompagni, Y Crow, A Martini, M Gattorno, P Picco

**Affiliations:** 1 G. Gaslini Institute, 2nd division of Pediatrics, Genova, Italy; 2 Manchester academic health science centre, Genetic Medicine, Manchester, UK; 3 Policlinico di Bari, Department of Pediatrics, Bari, Italy; 4 Universisty of Genova, department of Pediatrics, Genova, Italy

## Background

SAVI syndrome is a recently identified condition associated to mutations of TMEM173. Up to know only few cases of this disease have been described.

## Aim of the study

To describe the clinical manifestation of an Italian patient affected by SAVI syndrome.

## Results

The girl, first born from healthy, not relatives parents, at the age of 8 months started to present erythematosus-infiltrated skin lesions with pustular evolution and finally hesitating in scars in 15-20 days. From the age of three years chilblains and severe nail dystrophy appeared.

A CT scan performed at the age of 8 years revealed the presence of diffuse interstitial thickening with ground-glass appearance. A restrictive framework was detected at spirometry (FVC 51%).

The autoantibodies detection revealed positive ANA (1: 160), ANCA (1:80) and Coombs test.

The skin biopsy revealed a predominantly granulomatous nodular dermatitis, with folliculitis and secondary fibrosis.

The lung biopsy revealed focal hemorrhage, edema and predominantly lymphocytic inflammatory aggregates in the peribronchial interstitial areas with aspects of capillaritis and contiguous focal subatelettasia with alveolar cavity filled of macrophages.

Steroidal treatment (prednisone 1 mg/kg/day) was started with improvement of clinical manifestation, anemia and normalization of inflammatory markers. However attempts to reduce such therapy were followed by an exacerbation of the clinical picture.

Treatment with both immunosuppressive (azathioprine) and biologic (etanercept) drugs was tempted, without clear improvement. Unsatisfactory growth was also detected.

In the following months the child started to present a mild renal involvement with microscopic hematuria and hypertension, requiring anti-hypertensive treatment.

Given the evocative framework, interferon gene signature was performed, revealing a significant activation; the molecular analysis of TMEM173 gene showed the presence of the de novo Val155Met mutation, already described as causative of SAVI syndrome.

The child continued to present persistent severe microcytic anemia, requiring erythrocytes’ transfusions, despite high levels of erythropoietin. Bone marrow aspiration revealed dysmaturative signs in the in erythroid progenitors.

Treatment with jak1/2 inhibitor (Ruxolitinib, 5 mg day) was just started at the time of abstract presentation.

## Conclusion

This report of the first Italian patient with SAVI syndrome confirms the presence of the previously described clinical manifestations. Persistent hematuria and hypertension are reasonably signs of an underlying renal involvement, not previously described in this condition.

